# Maternal overweight and obesity and risk of pre-eclampsia in women with type 1 diabetes or type 2 diabetes

**DOI:** 10.1007/s00125-016-4035-z

**Published:** 2016-07-01

**Authors:** Martina Persson, Sven Cnattingius, Anna-Karin Wikström, Stefan Johansson

**Affiliations:** 1 Clinical Epidemiology Unit, Department of Medicine Solna, Karolinska Institutet, Eugeniahemmet T2, 17176 Stockholm, Sweden; 2 Department of Clinical Science and Education, Södersjukhuset, Karolinska Institutet, Stockholm, Sweden; 3 Department of Women’s and Children’s Health, Uppsala University, Uppsala, Sweden

**Keywords:** BMI, Diabetes type 1, Diabetes type 2, Moderate pre-eclampsia, Obesity, Overweight, Pre-eclampsia, Risk of pre-eclampsia, Severe pre-eclampsia

## Abstract

**Aims/hypothesis:**

Women with type 1 or type 2 diabetes are at increased risk of pre-eclampsia. Overweight and obesity are associated with an increased risk of pre-eclampsia in women without diabetes. The aim of the study was to investigate the impact of maternal overweight and obesity on the risk of pre-eclampsia in women with type 1 diabetes or type 2 diabetes.

**Methods:**

In a population-based cohort study including singleton births in Sweden, we estimated the risk of pre-eclampsia among women with type 1 diabetes (*n* = 7062) and type 2 diabetes (*n* = 886), and investigated whether maternal overweight (BMI 25–29.9 kg/m^2^) and obesity (BMI ≥30.0 kg/m^2^) modified the risk. Logistic regression analyses were used to estimate crude and adjusted ORs with 95% CIs, using women without diabetes as the reference group (*n* = 1,509,525).

**Results:**

Compared with women without diabetes, the adjusted ORs for pre-eclampsia in women with type 1 and type 2 diabetes were 5.74 (95% CI 5.31, 6.20) and 2.11 (95% CI 1.65, 2.70), respectively. The corresponding risks of pre-eclampsia combined with preterm birth were even higher. Risks of pre-eclampsia increased with maternal overweight (BMI 25–29.9 kg/m^2^) and obesity (BMI ≥30.0 kg/m^2^), foremost in women without diabetes, to a lesser extent in women with type 1 diabetes but not in women with type 2 diabetes.

**Conclusions/interpretation:**

Maternal overweight and obesity increased risks of pre-eclampsia in women with type 1 diabetes but not in women with type 2 diabetes. Even so, considering associations between maternal BMI and overall maternal and offspring risk, all women (with and without diabetes) should aim for a normal weight before pregnancy.

## Introduction

The outcome of pregnancies complicated by pregestational diabetes has markedly improved over the last few decades. However, the risk of severe maternal complications is still considerably increased [1, 2], including a 2–6 times higher risk of pre-eclampsia compared with women without diabetes [3]. Pre-eclampsia accounts for more than half of all maternal deaths per year in high income countries [4], and is associated with substantially increased risks of stillbirth, preterm birth and infant mortality [5, 6]. Effective predictors for its early detection are lacking [7] and delivery is the only cure for pre-eclampsia.

In women with type 1 diabetes, a number of risk factors for pre-eclampsia have been identified [8]. The most salient include elevated first trimester HbA_1c_ and both incipient and overt nephropathy [9, 10]. Risk factors for pre-eclampsia in women with type 2 diabetes are less well studied [11]. However, the prevalence of overweight and obesity continues to rise in general [12], also in women with type 1 or type 2 diabetes [13, 14].

In women without diabetes, high BMI is an important risk factor for both moderate and severe pre-eclampsia [15, 16]. We previously demonstrated that maternal overweight and obesity increase the risk of pre-eclampsia in women with type 1 diabetes [17]. However, we are unaware of studies focusing on associations between maternal overweight and obesity and the risk of pre-eclampsia in women with type 2 diabetes. Therefore, the aim of the current study was to compare the impact of maternal BMI on pre-eclampsia risks in women with type 1 and type 2 diabetes. We also wanted to explore the effect of maternal overweight and obesity on risks of pre-eclampsia subtypes, including moderate and severe pre-eclampsia, and pre-eclampsia leading to preterm birth.

## Methods

### Study design and study population

The national Swedish Medical Birth Register (MBR) contains data on more than 98% of all births in Sweden. Between 1997 and 2012, the MBR added information on 1,600,575 births. We excluded mothers with missing personal identification numbers (*n* = 19,001) and with no information on country of birth (*n* = 1108). We also excluded multiple births (*n* = 46,662) and births with unknown gestational age (*n* = 1122). In order to compare the risks of pre-eclampsia in women with type 1 and type 2 diabetes and women without diabetes, we also excluded women with gestational diabetes (*n* = 15,209). Thus, the final study population comprised 1,517,473 women with singleton births.

The Swedish MBR is subject to quality control by the National Board of Health and Welfare, and the coverage and validity of most variables are considered to be high [18]. Information on maternal sociodemographic factors, anthropometry, medical history, and obstetric and perinatal outcome is recorded prospectively by midwifes and physicians using standardised forms during pregnancy, delivery and the neonatal period. Maternal and neonatal diagnoses are classified by the physician according the International Classification of Diseases; ICD-10 has been used since 1997. We used the personal identification number [19] assigned to all citizens in Sweden to cross-link data from the MBR to the Total Population Register for the maternal country of birth and to the Education Register for the mother’s highest attained level of education [20, 21]. Ethical approval was obtained from the Research Ethics Committee of the Karolinska Institutet, Stockholm, Sweden (no. 2012/1813-31/4).

### Exposures

The main exposures were maternal type 1 and type 2 diabetes, and maternal BMI. Identification of women with type 1 or type 2 diabetes was based on ICD-10 codes O240 and O241, respectively. The reference group comprised women without gestational diabetes or type 1 or type 2 diabetes. Maternal BMI (kg/m^2^) was calculated from weight measured wearing light indoor clothing and self-reported height at the first antenatal visit, usually during the first trimester [18]. Women were categorised according to the WHO criteria as underweight (BMI <18.5 kg/m^2^), normal weight (BMI 18.5–24.9 kg/m^2^), overweight (BMI 25–29.9 kg/m^2^) or obese (BMI ≥30 kg/m^2^) [22].

### Covariates

Information on maternal height, age at delivery, parity and self-reported smoking, as recorded at the first antenatal visit, was retrieved from the MBR. Covariates were categorised as shown in Table 1.

**Table 1:** Maternal characteristics and rates of pre-eclampsia in women with singleton births in Sweden between 1997 and 2012

Maternal characteristics	Total (*n*)	Pre-eclampsia	
		*n*	%
Total	1,517,473	43,223	2.8
Maternal age (years)			
≤ 24	227,953	7602	3.3
25–29	469,675	13,521	2.9
30–34	523,845	13,448	2.6
≥ 35	296,000	8652	2.9
Parity			
Nulliparous	671,893	28,966	4.3
Parous	845,580	14,257	1.7
Early pregnancy BMI (kg/m^2^)			
< 18.5	32,095	538	1.7
18.5–24.9	830,141	17,661	2.1
25.0–29.9	338,897	11,340	3.4
30.0–34.9	106,178	5533	5.2
≥ 35.0	42,046	3213	7.6
Data missing	168,116	4938	2.9
Maternal height (cm)			
< 155	45,395	1429	3.2
155–164	499,906	15,094	3.0
165–174	737,640	20,348	2.8
≥ 175	142,284	3585	2.5
Data missing	92,248	2767	3.0
Daily smoking in early pregnancy			
No	1,308,648	38,040	2.9
Yes	128,841	2714	2.1
Data missing	79,984	2469	3.1
Education (years)			
9	376,896	10,714	2.8
10–11	394,385	12,604	3.2
12	210,515	5934	2.8
≥ 13	518,037	13,566	2.6
Data missing	17,640	405	2.3
Mother’s country of birth			
Nordic	1,243,543	37,592	3.0
Non-Nordic	273,930	5631	2.0

### Outcomes

The main outcome was pre-eclampsia, based on ICD-10 codes (O140, O141, O149 and O15). Pre-eclampsia was further divided into mild to moderate (ICD-10 codes O140 and O149, respectively) and severe (ICD-10 codes O141 and O15). Pre-eclampsia was also stratified by gestational age at delivery into preterm and term pre-eclampsia, defined as a recorded pre-eclampsia diagnosis combined with preterm or term delivery (at <37 weeks or ≥37 weeks, respectively). There are several definitions of pre-eclampsia, and classification may therefore vary between countries. In Sweden, the clinical definition of pre-eclampsia was BP ≥140/90 mmHg (systolic/diastolic) combined with proteinuria (at least 0.3 g per 24 h or a score of ≥1 on a urine dipstick on at least two subsequent occasions) during the study period. Severe pre-eclampsia is commonly defined as pre-eclampsia with a systolic BP ≥160 mmHg, and/or a diastolic BP ≥110 mmHg and/or multiorgan involvement [23]. A Swedish validation study found good agreement between registry information on pre-eclampsia diagnoses and information in the individual records when using ICD-9 codes [24], and a Danish study showed good validity for registry information on pre-eclampsia when using ICD-10 codes [25].

### Statistical analyses

Rates of pre-eclampsia were calculated separately for women without diabetes, women with type 1 diabetes and women with type 2 diabetes. We used logistic regression analyses to estimate ORs and 95% CIs for pre-eclampsia and subgroups of pre-eclampsia in women with type 1 or type 2 diabetes. Women without diabetes were used as the reference population. In the first adjusted model, ORs were only adjusted for maternal BMI (model 1), while in the second model ORs were additionally adjusted for maternal parity, age, height, smoking, maternal country of birth and education (model 2). To investigate whether the effect of BMI on pre-eclampsia risk differed in women with and without diabetes, we decided a priori to perform stratified analyses of associations between BMI and pre-eclampsia risk in women without diabetes, women with type 1 diabetes and women with type 2 diabetes. Interactions were tested separately for women with type 1 and type 2 diabetes. Interaction terms were introduced in the multivariate model (model 2), and a *p* value of <0.05 for the interaction term was considered statistically significant. For women with type 1 diabetes, analyses were stratified by BMI category for all subtypes of pre-eclampsia. Due to power concerns, it was not possible to perform these analyses for women with type 2 diabetes. In all analyses, the general estimation equation model was applied to account for repeated pregnancies.

## Results

Rates of pre-eclampsia were higher in younger (≤24 years) and nulliparous women, and increased with maternal BMI (Table 1). A lower pre-eclampsia rate was observed in smokers compared with non-smokers. Further, the rate of pre-eclampsia was higher in Nordic women than in non-Nordic women.

Compared with women without diabetes, the crude OR for pre-eclampsia was more than six times higher in women with type 1 diabetes and 3.5 times higher in women with type 2 diabetes. These risk estimates were reduced after adjustment for maternal BMI, especially for women with type 2 diabetes (Table 2, model 1). Further adjustment for other covariates only slightly changed these risks (Table 2, model 2).

**Table 2:** Type 1 and type 2 diabetes and risks of pre-eclampsia

Diabetic disease	Total (*n*)	Pre-eclampsia				
		*n*	%	Crude OR (95% CI)	Adjusted OR (95% CI)	
					Model 1^a^	Model 2^b^
No	1,509,525	42,034	2.8	1.00	1.00	1.00
Type 1	7062	1103	15.6	6.32 (5.88, 6.78)	5.74 (5.31, 6.20)	5.64 (5.19, 6.12)
Type 2	886	86	9.7	3.45 (2.71, 4.39)	2.11 (1.65, 2.70)	2.30 (1.67, 3.17)

^a^Adjusted for maternal BMI

^b^Adjusted for maternal BMI, height, age, parity, education, smoking and country of birth.

Type 1 and type 2 diabetes were both associated with increased ORs for all subgroups of pre-eclampsia, and type 1 diabetes was consistently associated with higher risks compared with type 2 diabetes (Table 3). Compared with women without diabetes, the adjusted OR for severe pre-eclampsia was almost six times higher in women with type 1 diabetes and approximately 2.5 times higher in women with type 2 diabetes. The highest ORs were observed for pre-eclampsia related to preterm birth. Compared with women without diabetes, the crude and adjusted ORs of pre-eclampsia with preterm birth were close to 11 times and nine times higher for women with type 1 diabetes. In women with type 2 diabetes, the OR for pre-eclampsia leading to preterm birth was reduced from 4.74 (95% CI 3.13, 7.16) to 3.11 (95% CI 2.05, 4.70) after adjustment for BMI (Table 3).

**Table 3:** Type 1 and type 2 diabetes and risks of pre-eclampsia subtypes

Pre-eclampsia subtype	Diabetic disease	Total (*n*)	Pre-eclampsia				
			*n*	%	Crude OR (95% CI)	Adjusted OR (95% CI)	
						Model 1^a^	Model 2^b^
Mild/moderate	No	1,509,525	28,460	1.9	1.00	1.00	1.00
	Type 1	7062	704	10.0	5.69 (5.24, 6.19)	5.08 (4.65, 5.56)	4.89 (4.45, 5.38)
	Type 2	886	58	6.5	3.44 (2.58, 4.58)	2.01 (1.50, 2.68)	2.18 (1.50, 3.16)
Severe	No	1,509,525	13,574	0.9	1.00	1.00	1.00
	Type 1	7062	399	5.6	6.51 (5.84, 7.25)	5.95 (5.29, 6.69)	5.75 (5.08, 6.52)
	Type 2	886	28	3.2	3.50 (2.37, 5.16)	2.29 (1.53, 3.45)	2.45 (1.47, 4.07)
Preterm (<37 weeks)	No	1,509,525	9360	0.6	1.00	1.00	1.00
	Type 1	7062	435	6.2	10.35 (9.29, 11.52)	9.08 (8.06, 10.22)	8.72 (7.68, 9.89)
	Type 2	886	27	3.1	4.74 (3.13, 7.16)	3.11 (2.05, 4.70)	3.22 (1.93, 5.37)
Term (≥37 weeks)	No	1,509,525	32,674	2.2	1.00	1.00	1.00
	Type 1	7062	668	9.5	4.65 (4.27, 5.06)	4.30 (3.93, 4.71)	4.19 (3.81, 4.62)
	Type 2	886	59	6.7	3.06 (2.32, 4.03)	1.80 (1.36, 2.40)	1.96 (1.36, 2.82)

^a^Adjusted for maternal BMI (kg/m^2^)

^b^Adjusted for maternal BMI, height, age, parity, education, smoking and country of birth

Data on BMI was available for 88.3% of women with type 1 diabetes, 90.6% of women with type 2 diabetes and 88.9% of women in the reference population. In all, 0.4% of women with type 1 diabetes were underweight, 47.9% were normal weight, 33.6% were overweight and 18% were obese. Corresponding figures for women with type 2 diabetes were: underweight, 0.5%; normal weight, 16.9%; overweight, 27.7%; and obese, 54.7%. There were significant interactions between maternal BMI and diabetes status with regard to pre-eclampsia risk for both type 1 (*p* < 0.001) and type 2 (*p* = 0.01) diabetes. We therefore performed stratified analyses of associations between maternal BMI and pre-eclampsia in women without diabetes, with type 1 diabetes, and with type 2 diabetes. Especially in women without diabetes, but also in women with type 1 diabetes, rates of pre-eclampsia increased with maternal overweight and obesity (Table 4). The highest overall pre-eclampsia rate (18.6%) was seen in obese women with type 1 diabetes. For those without diabetes, overweight and obese women had 1.7-fold and a more than threefold increased odds of pre-eclampsia, respectively, compared with normal weight women (Table 4). In women with type 2 diabetes, overweight and obesity were not associated with increased risks of pre-eclampsia compared with normal weight.

**Table 4:** Risks of pre-eclampsia stratified by maternal BMI in women with and without diabetes

Diabetic disease	BMI (kg/m^2^)					
	18.5–24.9		25–29.9		≥30	
	%	OR (95% CI)^a^	%	OR (95% CI) ^a^	%	OR (95% CI) ^a^
No	2.1	1.00	3.3	1.69 (1.64, 1.73)	5.8	3.22 (3.12, 3.32)
Type 1	14.2	1.00	16.1	1.17 (0.99, 1.38)	18.6	1.48 (1.20, 1.82)
Type 2	8.8	1.00	6.9	0.84 (0.33, 2.16)	11.5	1.23 (0.56, 2.72)

^a^Underweight women were excluded

^b^Adjusted for maternal BMI, height, age, parity, education, smoking and country of birth

Compared with normal weight women with type 1 diabetes, obesity led to modest increments in the risk of all subtypes of pre-eclampsia: the adjusted ORs for moderate pre-eclampsia, severe pre-eclampsia and pre-eclampsia with preterm birth in obese women with type 1 diabetes were 1.42 (95% CI 1.11, 1.82), 1.49 (95% CI 1.09, 2.05) and 1.33 (95% CI 0.97, 1.83), respectively.

## Discussion

To our knowledge, the present population-based cohort study is the first to demonstrate the impact of maternal BMI in women with type 1 and type 2 diabetes on the risk of pre-eclampsia, including subtypes of pre-eclampsia. We found no evidence that maternal overweight and obesity are associated with an increased risk of pre-eclampsia in women with type 2 diabetes, as opposed to women with type 1 diabetes and, especially, women without diabetes. The observation that rates of pre-eclampsia were increased in women with type 1 and type 2 diabetes is in accordance with previous reports. However, we also found consistently higher rates of all subtypes of pre-eclampsia in women with type 1 diabetes compared with women with type 2 diabetes. Rates of pre-eclampsia were also significantly higher in Nordic than in non-Nordic women. This is consistent with previous findings [26, 27].

Compared with women without diabetes, the ORs for pre-eclampsia and subtypes of pre-eclampsia were substantially increased in women with type 1 diabetes, and risk estimates were only slightly attenuated after adjustment for BMI. This demonstrates that the association between type 1 diabetes and pre-eclampsia is independent of BMI, even though the odds for pre-eclampsia increased further with maternal overweight and obesity. In contrast, in women with type 2 diabetes, the risk of pre-eclampsia was partially confounded by BMI, while increasing maternal BMI did not further increase the pre-eclampsia risk. These discrepancies probably reflect differences in genetic factors and the clinical expression of both type 1 and type 2 diabetes. In addition, the impact of BMI on the risk of pre-eclampsia may vary with the duration of diabetes, and the presence or absence of diabetes-related complications and other risk factors for pre-eclampsia. The lack of effect of BMI on the risk of pre-eclampsia in women with type 2 diabetes and the modestly increment in pre-eclampsia risk conveyed by maternal obesity in women with type 1 diabetes demonstrate that diabetes disease per se is a much stronger risk factor for pre-eclampsia than BMI. This is also consistent with comparable risks of cardiovascular death for normal weight and overweight/obese adults with type 2 diabetes [28]. However, the vast majority (83%) of women with type 2 diabetes in our study were either overweight or obese (BMI ≥25 kg/m^2^), and considerably fewer women with type 2 diabetes than with type 1 diabetes. Thus, it is possible that the range of exposures was insufficient to demonstrate a significant contribution of BMI on the risk of pre-eclampsia in women with type 2 diabetes or that a larger cohort was needed to demonstrate an effect.

Studies comparing rates of pre-eclampsia in women with type 1 diabetes and type 2 diabetes are scarce, and contradictory results have been reported [29, 30]. In the present study, rates of pre-eclampsia and subtypes of pre-eclampsia were significantly higher in women with type 1 diabetes than in women with type 2 diabetes. Differences in the rates of pre-eclampsia in women with type 1 diabetes or type 2 diabetes may be attributable to differences in risk factors, such as chronic hypertension, level of glycaemic control, duration of diabetes disease and diabetes microangiopathy. In addition, comparison of placental findings in women with type 1 or type 2 diabetes suggest that the strain of pregnancy may affect maternal vascularisation differently in these two groups [31].

Diabetic nephropathy is a complication of around 2.5–5% of pregnancies in women with diabetes [32, 33] and is a strong risk factor for pre-eclampsia [34]. Interestingly, in women with type 1 diabetes and nephropathy, maternal overweight was the only additional factor associated with adverse pregnancy outcome, whereas duration of diabetes, level of glycaemic control and weight gain in pregnancy did not influence risks [35]. Microalbuminuria, the forerunner to diabetic nephropathy has also been associated with a fourfold increased risk of pre-eclampsia in women with type 1 diabetes [36]. There has been less investigation into the impact of different degrees of nephropathy on the risk of pre-eclampsia in women with type 2 diabetes. However, a recent nationwide study from Denmark demonstrated that women with type 2 diabetes and overt nephropathy or microalbuminuria had comparable rates of pre-eclampsia to those of women with type 1 diabetes and different degrees of diabetic nephropathy [32]. Elevated HbA_1c_ in early pregnancy is another important risk factor for pre-eclampsia in women with type 1 diabetes [9, 37]. Obesity is associated with increased insulin resistance and elevated glucose concentrations [38]. Against this background, one might speculate that the increment in risk of pre-eclampsia in obese women with type 1 diabetes may reflect a poorer glycaemic control compared with normal weight women with type 1 diabetes. Furthermore, the aetiology of pre-eclampsia remains undefined. Pre-eclampsia includes abnormal vascular development of the placenta and systemic maternal endothelial dysfunction [3]. Obesity and pre-eclampsia share several features, including subclinical inflammation, insulin resistance and lipotoxicity [39, 40]. Lipotoxicity is associated with maternal vascular dysfunction and reduced trophoblast invasion into the developing placenta; it has been proposed to link maternal overweight and obesity to the risk of pre-eclampsia [39]. It has also been hypothesised that maternal obesity may increase the risk of pre-eclampsia by reducing endogenous production of nitric oxide, with the subsequent increased risk of endothelial dysfunction [41]. Adverse metabolic and vascular effects of overweight and obesity are particularly seen in visceral obesity. Thus, it is possible that the impact of maternal BMI on the risk of pre-eclampsia may differ between women with different fat mass distributions.

Strengths of this study include the population-based design, including data on all pregnancies in women with type 1 diabetes and type 2 diabetes in Sweden between 1997 and 2012. Data were prospectively collected within a healthcare context, which largely excludes recall bias. The large study cohort enabled us to investigate risks of pre-eclampsia and subtypes of pre-eclampsia in women with type 1 diabetes and type 2 diabetes, and also to investigate the importance of BMI in stratified analyses. In Sweden, management of pregnancies with pregestational diabetes is uniform and pregnancy outcomes do not differ with geographical area [1]. We were able to adjust for important maternal diagnoses and confounders [18, 24, 25]. During the study period, the main screening strategy for identifying gestational diabetes was repeated capillary random plasma glucose values of ≥9 mmol/l or the presence of traditional risk factors (i.e. first-degree family history of diabetes, obesity, history of gestational diabetes or delivery of a large-for-date baby in a previous pregnancy). Plasma glucose is also measured in cases of glucosuria. In addition, an OGTT is usually also performed in cases of fetal growth acceleration or polyhydramnios. A diagnosis of gestational diabetes was based on an OGTT (75 g glucose) performed within a week for cases in which a random plasma glucose value was above the limit. Women with traditional risk factors are tested once or twice during the first 30 weeks of gestation. A diagnosis of gestational diabetes mellitus was based on a fasting plasma glucose value of ≥7 mmol/l and/or a 2 h plasma glucose value of ≥10 mmol/l. As almost all obese women (BMI ≥30 kg/m^2^) are submitted to an OGTT during antenatal care, it is unlikely that gestational diabetes was undetected in this group of women.

While we consider the risk of misclassification of type 1 diabetes to be negligible, we cannot exclude the possibility that some misclassification of type 2 diabetes and gestational diabetes may have biased our results: women with type 2 diabetes may have been misclassified as having gestational diabetes.

The Swedish MBR does not contain information on maternal glycaemic control, duration of diabetes or diabetes-associated complications. In this study, we used BMI in early pregnancy as a proxy for fat mass. The validity of this proxy measure is supported by the strong association (*r*^2^ = 0.84) between BMI and total fat mass in early pregnancy [42]. However, it is important to remember that the impact of maternal BMI on the risk of pre-eclampsia may differ with distribution of fat mass.

In conclusion, we found that maternal overweight and obesity increase the risks of pre-eclampsia and subtypes of pre-eclampsia in women with type 1 diabetes and, especially, in women without diabetes. In contrast, maternal BMI did not substantially influence the risk of pre-eclampsia in women with type 2 diabetes. Our findings indicate that striving for a normal pre-pregnancy BMI could reduce the risk of pre-eclampsia in women with type 1 diabetes and women without diabetes. Further, as maternal BMI is also related to other pregnancy and obstetric complications, women with type 2 diabetes would probably benefit in other respects from having a normal weight. Given the increasing prevalence of maternal obesity and the severity of the preeclamptic disease, it is important that our results are confirmed in other populations.

## Abbreviation

MBR: Medical Birth Register
